# 

**Published:** 1902-11-08

**Authors:** 


					sihe hospital. "The standard of highest purity."?Lancet. Nov. 8th, 1902.
CADBURY'S ^ CADBURY'S
COCOA H 0000A
ABSOLUTELY PURE. j#? ? ? -7- MO DRUGS OR ALKALIES USES.
No. 84 lr Yol. XXXIII. [fftwe"] Saturday,
Nov. 8th, 1902.
[Sub80rsepetp0n9?nns'] PRICE TWOPENCE

				

